# The Relationship between Subjective Visual Impairment and Domain‐Specific Cognitive Performance in Cognitively Normal Older Adults and MCI Patients

**DOI:** 10.1002/alz.090778

**Published:** 2025-01-09

**Authors:** Jordan P Sergio, Abigail Dubois, Louisa I. Thompson, Jennifer Strenger, Ashley N. Price, Megan Stradtman, Stuart Sinoff, Peter J. Snyder, Jessica Alber

**Affiliations:** ^1^ University of Rhode Island, Kingston, RI USA; ^2^ Butler Hospital Memory and Aging Program, Providence, RI USA; ^3^ Alpert Medical School of Brown University, Providence, RI USA; ^4^ Bryn Mawr College, Bryn Mawr, PA USA; ^5^ BayCare Health, Clearwater, FL USA

## Abstract

**Background:**

Age is the greatest risk factor for self‐reported visual impairment (VI) and Alzheimer’s Disease (AD). While prior studies demonstrate associations between VI and cognitive screening measures in older adults, the relationship between VI and specific cognitive domains remains unexplored. We examined the relationship between subjective VI and executive function, attention, visuospatial construction, episodic memory, and processing speed.

**Method:**

Participants were older adults aged 55‐80: 58 cognitively unimpaired (CU) (MoCA>=26, CDR=0) and 19 MCI patients (MoCA>19, <24, CDR=.5). Subjective VI was measured with the National Eye Institute Visual Function Questionnaire (NEI‐VFQ). A neuropsychological battery including the MoCA, CDR, Digit Symbol Substitution Test (DSST), Repeatable Battery for the Assessment of Neuropsychological Status (RBANS), and Free and Cued Selective Reminding Test (FCSRT) was administered to assess cognition. Partial correlations controlling for age and years of education compared subjective VI scores to cognitive scores. A multivariate, repeated measures ANCOVA was run on a subset (N=23) of participants with 1 year follow‐up data to determine whether group performance differed over time on NEI‐VFQ and DSST.

**Result:**

There were significant correlations between scores on the composite NEI‐VFQ and both the DSST and visuospatial sub‐scale of the RBANS in the CU and patient groups (p<.05). CU adults showed a greater practice effect over time on the DSST than the patient group, who declined in performance. Specifically, CU APOE E4 carriers had a larger increase in DSST score over 12 months than non‐carriers, along with a larger increase in subjective VI on the NEI‐VFQ.

**Conclusion:**

Attention, processing speed, visuospatial construction, and executive function performance was related to a standardized measure of subjective VI in CU older adults and MCI patients. After 12 months, CU APOE E4 carriers showed a stronger practice effect than CU non‐ carriers on the DSST, concomitant with increase in subjective VI. Subjective measures of VI may serve as a tool in early detection of cognitive impairment, although increases in VI over time co‐occurred with increased practice effects in CU APOE E4 carriers. Longitudinal studies are ongoing to examine the predictive value of subjective VI on cognitive impairment.

